# Assessment of the antinociceptive and anti-inflammatory activities of the stem methanol extract of *Diplotropis purpurea*

**DOI:** 10.1080/13880209.2019.1628074

**Published:** 2019-06-26

**Authors:** Lorena A. Cruz, Miguel A. Díaz, Mahabir P. Gupta, José Luis López-Pérez, Eily Mondolis, Juan Morán-Pinzón, Estela Guerrero

**Affiliations:** a Dirección Nacional de Farmacia y Drogas, Ministerio de salud, Panama, Panama;; b Centro de Estudios Farmacognósticos de la Flora Panameña, Facultad de Farmacia, Universidad de Panamá, Panama, Panama;; c Departamento de Farmacología, Escuela de Medicina, Universidad de Panamá, Panama, Panama;; d Departmento de Ciencias Farmacéuticas, IBSAL-CIETUS, Universidad de Salamanca, Salamanca, Spain

**Keywords:** Fabaceae, carrageenan, paw oedema, hot plate, formalin test, writhing test

## Abstract

**Context:** Since there is still a great need to search for plant species with antinociceptive and anti-inflammatory activities, *Diploptropis purpurea* (Rich.) Amshoff (Fabaceae) is studied for the first time.

**Objective:** This evaluates the analgesic and anti-inflammatory activities of the stem methanol extract of *Diplotropis purpurea* (MEDP).

**Material and methods:** The anti-inflammatory and analgesic effects of MEDP of *D. purpurea* were evaluated *in vivo*. The antinociceptive activity was assessed in CD1 male mice were treated by oral gavage with 500 mg/kg of MEDP 30 min before submitting to acetic acid-induced abdominal writhing, hot-plate, and formalin tests. Paws oedema induced by carrageenan, histamine or serotonin were performed in male Sprague–Dawley rats to determinate the anti-inflammatory activity.

**Results:** Oral administration of MEDP produced significant antinociceptive effects on the inflammatory phase in the formalin test [12.0 s versus 72.5 s in carboxymethyl cellulose (CMC) control group]. MEDP produced an analgesic effect in the hot-plate model, although the effect was modest compared to tramadol (40 and 60%, respectively). The oral administration of MEDP in a dose of 500 mg/kg showed maximum inhibition (75.1%) after 0.5 h in carrageenan-induced oedema, but it did not modify histamine or serotonin-induced oedemas.

**Discussion and conclusion:** In the peripheral nociception model, acetic acid-induced abdominal writhing, the MEDP did not show a protective effect, but its analgesic effects were evident in the inflammatory phase of the formalin test and in the hot-plate model. These results show that the anti-inflammatory effect was accompanied by a reduction in the perception of painful stimuli.

## Introduction

The Center for Pharmacognostic Research on Panamanian Flora (CIFLORPAN) represents one of the most important institutions at the regional level dedicated to the study of Panamanian flora whose potential has not yet been fully explored. Since its foundation, this Center has led a number of phytochemical and pharmacological studies related to Panamanian flora (Caballero-George and Gupta 2011). Currently, several investigations are underway, one of which deals with the study of plants of the Fabaceae family.

*Diploptropis purpurea* (Rich.) Amshoff (Fabaceae), selected as part of a bioprospecting study conducted by CIFLORPAN, is a plant that has no reported ethnobotanical uses, but it shows antiprolifereative activity (Olmedo et al. 2016). It has a geographical distribution that extends from Panama to Brazil, where it is popularly known as ‘*supupira’*. Previous phytochemical studies have described the presence of constituents for which studies of analgesic and antioxidant activity have been reported (Braz Filho et al. 1973; Geetha and Varalakshmi 2001; Alrushaid et al. 2016), among others. However, this plant has not been subjected to previous pharmacological or toxicity studies. Therefore, we decided to evaluate the properties of the MEDP.

## Materials and methods

### Plant material and preparation of plant extract

The stems of *D. purpurea* were collected in July 2008 from Agua Clara, Sierra Llorona, Santa Rita, Colon Province, Panama, and identified by Alex Espinosa, taxonomist of CIFLORPAN. A voucher specimen Florpan 7951A has been deposited at the Herbarium of the University of Panama (PMA). The plant material was air-dried and pulverized in a Wiley mill. The powdered plant material (100 g) was extracted twice (for 24 h) by maceration in methanol and concentrated *in vacuo* using rotary evaporator at low temperature (<40 °C) yielding a brown residue denominated as MEDP.

### Experimental animals

Experiments were carried out using adult male CD1 mice (18–25 g) and adult male Sprague–Dawley rats (150–200 g), obtained from the Animal House of the Faculty of Veterinary Medicine, University of Panama. Animals were maintained under standard room conditions (temperature 22 ± 2 °C and relative humidity 55 ± 5 °C with 12 h light/dark cycle for 7 d before the experiment) with standard rodent diet and water *ad libitum.* When necessary, animals were deprived of food 12 h prior to the experiments. All experimental procedures followed the ‘Guidelines for the Care and Use of Laboratory Animals’ of the National Research Council (NRC), of the National Academies (NRC 2011). A high dose of pentobarbital was administered for euthanasia. Prior authorization for the use of laboratory animals in this study was obtained from the Bioethics Committee of the Pharmacology Department of School of Medicine (CBF-02DEC11).

### Acute toxicity study

This study was performed according to the Organization for Economic Co-operation (OECD) Guidelines (OECD 2001). Rats were divided into three groups of eight animals each. Different doses (500, 1000 and 2000 mg/kg) of MEDP were administered by oral gavage. Later the animals were observed for 24 h (0.5, 1, 3, 6 and 12 h) and daily until day 14 after dosing.

### Assessments of the antinociceptive activity

#### Acetic acid-induced abdominal writhing test

The writhing test was performed using a slightly modified method described by Cidade et al. (2016). Briefly, three groups of mice (*n* = 6) were orally pretreated with MEDP (500 mg/kg), acetylsalicylic acid (ASA), (200 mg/kg) or vehicle (CMC), (200 mg/kg). Thirty-five minutes later each mouse was exposed to acetic acid (10 mL/kg; IP), and the number of writhings per mouse were counted for 30 min.

#### Formalin test

Mice were pretreated orally with MEDP (500 mg/kg), ASA (200 mg/kg) or CMC (200 mg/kg) or tramadol (20 mg/kg; s.c.). Thirty minutes later, each mouse received an intra-plantar injection of formalin (20 µL; 1.4%). The duration of paw licking was recorded at the early phase or neurogenic pain (1–5 min) and late phase or inflammatory pain (15–30 min) after formalin injection (Hunskaar and Hole 1987).

#### Hot plate test

Animals were divided into different groups and pretreated orally with MEDP (500 mg/kg), CMC (200 mg/kg) or tramadol (20 mg/kg, s.c.). Thirty minutes after each treatment, mice were individually placed on the hot plate (Socrel^^®^^ DS-37) setting at 55 ± 0.2 °C, to determine the reaction time (Lino et al. 2017).

### Assessments of anti-inflammatory activity

#### Carrageenan-induced paw oedema

Male rats were randomly divided into three groups (*n* = 6 per group) and treated by oral gavage with MEDP (500 mg/kg), indomethacin (INDO, 10 mg/kg) or CMC (0.1 mL/100 g). One hour after the administration, acute paw oedema was induced by subplantar injection of λ-carrageenan (0.1 mL, 1%, w/v), in a preventive model. A second group of experiments were conducted to evaluate the curative effect of MEDP. In the curative model, λ-carrageenan was administered 1 h before the treatment. Paw volumes were measured by Plethysmometer (Panlab Harvard Apparatus^®^ LE7500) at 0.5 h and every hour up to 6 h afterwards. In the preventive model, the measurements were made at 4, 4.5, 5, 6 and 24 h after carrageenan injection (Li et al. 2017).

#### Histamine and serotonin-induced paw oedema

The anti-inflammatory activity of the MEDP was evaluated according to the method previously described (Shabbir et al. 2018). The paw oedema was induced by sub-plantar administration of 0.1 mL of freshly prepared solutions of histamine (0.5%) or serotonin (5-HT) (0.5%). The paw volumes were recorded at 0, 0.5, 1 and 2 h after administration of inflammatory drug. Rats were pretreated orally with MEDP (500 mg/kg) or CMC, 1 h before inducing paw oedema. Loratadine (10 mg/kg) and cyproheptadine (10 mg/kg) were used as standard drugs against histamine and 5-HT induced oedema, respectively.

### Statistical analysis

Data obtained from animal experiments were expressed as the mean ± standard error of the mean (SEM). Statistical differences between the treated and the control groups were analyzed statistically by one-way ANOVA followed by Dunnet’s post-test or two-way ANOVA followed by Bonferroni post-test. All data were processed with GraphPad Prism 5.01 Software (GraphPad Software, La Jolla, CA).

## Results

### Acute toxicity study

In the acute toxicity study, MEDP administered in the highest dose (2000 mg/kg) did not produce mortality. No significant behavioural changes were observed after 48 h., which led us to conclude that the MEDP did not show any signs of toxicity after acute administration in rats. Based on this, a dose of 500 mg/kg of extract was selected for further studies.

### Assessments of the antinociceptive activity

#### Acetic acid-induced abdominal writhing test

**Figure 1.**
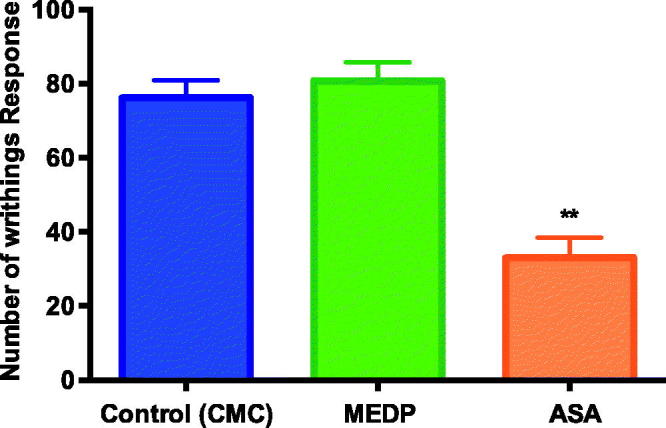
Effect of methanol extract of *D. purpurea* (MEDP) on acetic acid-induced abdominal writhing in mice. **p < 0.01 statistically significant compared to CMC group.

The analgesic activity of the MEDP was initially evaluated using the acetic acid-induced abdominal writhing test. Positive control group, which received ASA (200 mg/kg), showed a 56.7% analgesic effect. However, treatment with MEDP did not generate a significant protective effect against the algesic stimulus produced by the administration of acetic acid (Figure 1).

#### Formalin test

**Figure 2.**
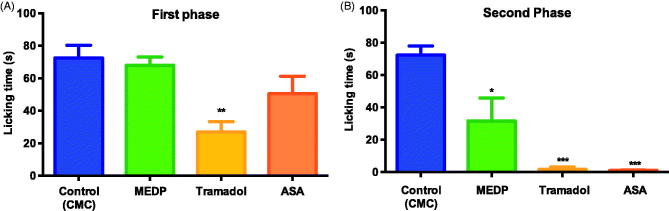
Effect of methanol extract of *D. purpurea* (MEDP) in formalin induced paw-licking test on first phase (A) and second phase (B) in mice. Each column represents the mean ± SEM of 6 mice. *p < 0.05; **p < 0.01; ***p < 0.001 statistically significant compared to the CMC group.

In the negative control group (CMC), an average licking time of 72.5 s during the neurogenic phase of the formalin test (0–5 min) was observed (Figure 2(A)). A reduction in licking time was seen in the tramadol group (26.94 s). However, in this first phase, neither ASA nor MEDP significantly modified the nociceptive-induced response, with a licking time of 50.4 and 67.9 s, respectively. The second phase of this test, indicative of inflammatory pain (15–30 min), was marked by the efficacy of both the standard compounds ASA and tramadol, as well as MEDP (1.1, 1.6 and 12.0 s, respectively) versus the licking time of 72.4 s recorded in the CMC control group (Figure 2(B)).

#### Hot-plate test

To assess the antinociceptive actions of the MEDP, the hot plate test was used. Tramadol increased the latency time of heat perception, resulting in analgesic percentages higher than 60% (Table 1). Also, the administration of MEDP produced an increase in latency time in the hot plate model, although the effect was modest compared to tramadol. A maximum analgesic activity of 39.6% was observed at 60 min.

**Table 1.** Reaction time (sec) obtained on hot plate test in mice.

Group	Periods of observation (min)			
	0	30	60	120
CMC	5.6 ± 0.5	6.9 ± 0.76	6.4 ± 0.6	8.0 ± 0.9
MEDP	7.3 ± 1.8	11.1 ± 1.5*	12.3 ± 2.3*	11.0 ± 1.7
		(30.0 %)	(39.6 %)	(29.1%)
Tramadol	5.7 ± 0.8	14.3 ± 1.9**	14.3 ± 2.2**	15.0 ± 1.9**
		(60.1%)	(60.4 %)	(65.2 %)

Results expressed as the mean ± SEM, *n* = 6 animals in each group. Statistical differences between groups were analyzed statistically by one-way ANOVA. *p < 0.05 and **p < 0.01 compared with CMC.

### Assessments of anti-inflammatory activity

#### Carrageenan-induced paw oedema

**Figure 3.**
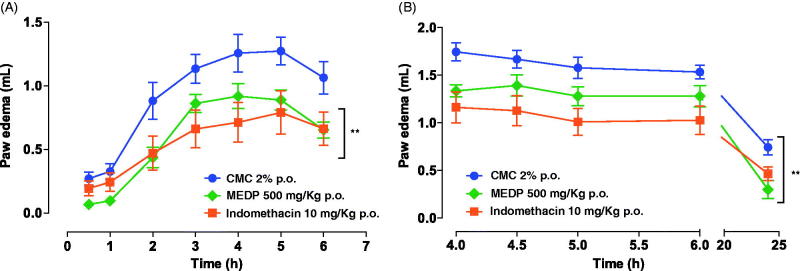
Effect of methanol extract of *D. purpurea* (MEDP) in paw oedema induced by carrageenan in rats (preventive model (A) and curative model (B)). Each group of treatment represents the mean ± SEM of six rats. **p < 0.01 statistically significant compared to the CMC group.

The carrageenan, histamine and 5-HT-induced paw oedemas were used to assess the anti-inflammatory properties of MEDP. During the first 30 and 60 min after carrageenan administration, animals treated with MEDP caused an inhibitory effect (75.1 and 70.7%, respectively). Although in the following periods, the protective effect was less noticeable, the ability of the extract to reduce the inflammatory effect was more significant. The volume displaced in the MEDP group was 0.4 and 0.6 mL, at 2nd and 6th hour, respectively, meanwhile, the CMC control group developed oedema volume of 0.8 and 1.1 mL at the same periods, respectively. Anti-inflammatory effects observed in indomethacin group corroborate the validity of our results. In these animals, the anti-inflammatory activity was visible from the second hour of observation (0.5 mL) to the last record obtained at the 6th hour (0.7 mL), values representing an inhibition of 46.7 and 37.7%, for each observation period (Figure 3(A)).

The modified oedema test was conducted to quantify curative anti-inflammatory effects of the MEDP. The treatment with indomethacin had a protective effect of over 32% at all observation times (4–24 h). For the group that received MEDP, all the determinations were statistically significantly lower (*p* < 0.01) than the CMC control group, being especially noteworthy the values observed at 4 h (1.3 mL; 23.5% anti-inflammatory effect) and 24 h (0.3 mL; 59.6% anti-inflammatory effect). These results were statistically significantly different (*p* < 0.01) compared to CMC control group (1.7 and 0.7 mL at 4 and 24 h, respectively) (Figure 3(B)).

#### Histamine and serotonin-induced paw oedema

**Figure 4.**
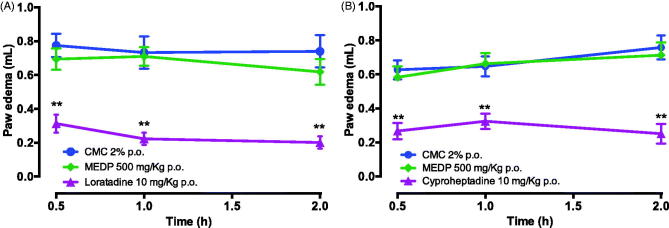
Effect of methanol extract of *D. purpurea* (MEDP) in paw oedema induced by histamine (A) and 5-HT (B) in rats. Each group of treatment represents the mean ± SEM of six rats. **p < 0.01 statistically significant compared to the CMC group.

For both histamine and serotonin-induced paw oedema models, standard drugs, loratadine and cyproheptadine produced a significant reduction in edema at all observation times. Reduction was 72.8% for loratadine and 66.9% for cyproheptadine. The extract did not attenuate histamine (Figure 4(A)) or 5-HT-induced paw oedema (Figure 4(B)).

## Discussion

The administration of MEDP in the pain-inflammation, formalin and carrageenan (curative and preventive) models, show a clear evidence of the capacity of this plant extract to control the inflammatory and nociceptive processes. Considering that the best results in our study were obtained in carrageenan-induced oedema and phase 2 of the formalin test, it can be assumed that the analgesic and anti-inflammatory effects of the MEDP could be associated with the inhibition of prostaglandin synthesis, since prostaglandins are known to be the main mediators responsible for inflammation and pain. In addition, the anti-inflammatory properties found for this plant do not appear to be determined by its ability to modulate neither the actions of histamine nor 5-HT.

Previous phytochemical studies revealed the presence of several bioactive constituents in *D. purpurea*: β-sitosterol, stigmasterol, lupeol, liquiritigenin [(±)-7,4′-dihydroxyflavanone], (–)-maackiain [(6aR,11aR)-3-hydroxy-8,9-methylenedioxyperocarbons], (2*R*)-7-hydroxyflavanone, formononetin and isoliquiritigenin (4,2′,4-trihydroxychalcone) (Braz Filho et al. 1973). It can be expected that the inhibitory response to nociceptive stimuli, as well as inflammatory response shown by the MEDP in our study, could be related to the properties described for its constituents. For example, an interesting study reported the effectiveness of lupeol for treating or reducing inflammation in a rat arthritis model (Geetha and Varalakshmi 2001). Formonetine in cell cultures has demonstrated potent anti-inflammatory properties, due to its ability to reduce the release of histamine (Xu and An 2017). In addition, anti-inflammatory and antinociceptive effects are reported for isoliquirititigenin and liquirititigenin (Kim et al. 2008; Shi et al. 2012), as well as other properties such as oxytocic, anti-diabetic, and hepatoprotective effects (Gaur et al. 2010; Gaur et al. 2014; Alrushaid et al. 2016).

In view of the above, it can be concluded that the inhibitory response to nociceptive stimuli, as well as the inhibition of the inflammatory response shown by the MEDP in this study, could be related to the properties described for its constituents. In addition, a recent study on MEDP showed antiproliferative activity against prostate and breast cancer cell lines (Olmedo et al. 2016). Since that excessive cell proliferation has been related to inflammatory processes (Crunkhorn and Meacock 1971; Schetter et al. 2010), the results in our study, as well as those reported for the constituents of this plant, highlight its ability to control inflammation, which may be due to the above reported antiproliferative activity.

Due to the results of pharmacological activity obtained in this preliminary study, an exhaustive bioguided phytochemical study of this plant, almost half a century after the unique study reported by Braz Filho et al. (1973) would be desirable since the substantial improvement in structural identification techniques, especially NMR, would enable the identification of a greater number and possibly new bioactive compounds responsible for the effects reported in this study.

## References

[CIT0001] Alrushaid S, Davies NM, Martinez SE, Sayre CL 2016 Pharmacological characterization of liquiritigenin, a chiral flavonoid in licorice. Res Pharm Sci. 11:355–365. 27920817 10.4103/1735-5362.192484 PMC5122824

[CIT0002] Braz Filho R, Gottlieb O, Vieira Pinho S, Queiroz Monte FJ, Da Rocha AI 1973 Flavonoids from Amazonian Leguminosae. Phytochemistry. 12:1184–1186.

[CIT0003] Caballero-George C, Gupta MP 2011 A quarter century of pharmacognostic research on Panamanian flora: a review. Planta Med. 77:1189–1202. 21674433 10.1055/s-0030-1271187

[CIT0004] Cidade AF, Vasconcelos PA, Silva DPB, Florentino IF, Vasconcelos GA, Vaz BG, Costa EA, Liao LM, Menegatti R 2016 Design, synthesis and pharmacological evaluation of new anti-inflammatory compounds. Eur J Pharmacol. 791:195–204. 27590355 10.1016/j.ejphar.2016.08.033

[CIT0005] Crunkhorn P, Meacock SC 1971 Mediators of the inflammation induced in the rat paw by carrageenin. Br J Pharmacol. 42:392–402. 4104654 10.1111/j.1476-5381.1971.tb07124.x PMC1665672

[CIT0006] Gaur R, Kumar S, Trivedi P, Bhakuni RS, Bawankule DU, Pal A, Shanker K 2010 Liquiritigenin derivatives and their hepatotoprotective activity. Nat Prod Commun. 5:1243–1246. 20839627

[CIT0007] Gaur R, Yadav KS, Verma RK, Yadav NP, Bhakuni RS 2014 *In vivo* anti-diabetic activity of derivatives of isoliquiritigenin and liquiritigenin. Phytomed: Int J Phytother Phytopharmacol. 21:415–422. 10.1016/j.phymed.2013.10.015 24262065

[CIT0008] Geetha T, Varalakshmi P 2001 Anti-inflammatory activity of lupeol and lupeol linoleate in rats. J Ethnopharmacol. 76:77–80. 11378285 10.1016/s0378-8741(01)00175-1

[CIT0009] Hunskaar S, Hole K 1987 The formalin test in mice: dissociation between inflammatory and non-inflammatory pain. Pain. 30:103–114. 3614974 10.1016/0304-3959(87)90088-1

[CIT0010] Kim YW, Zhao RJ, Park SJ, Lee JR, Cho IJ, Yang CH, Kim SG, Kim SC 2008 Anti-inflammatory effects of liquiritigenin as a consequence of the inhibition of NF-kappa B-dependent iNOS and proinflammatory cytokines production. Br J Pharmacol. 154:165–173. 18332856 10.1038/bjp.2008.79 PMC2438972

[CIT0011] Li X, Li N, Sui Z, Bi K, Li Z 2017 An Investigation on the quantitative structure-activity relationships of the anti-inflammatory activity of diterpenoid alkaloids. Molecules. 22:363–277. 10.3390/molecules22030363 PMC6155234 28264454

[CIT0012] Lino RC, da Silva DPB, Florentino IF, da Silva DM, Martins JLR, Batista DDC, Leite KCS, Villavicencio B, Vasconcelos GA, Silva ALP, et al. 2017 Pharmacological evaluation and molecular docking of new di-tert-butylphenol compound, LQFM-091, a new dual 5-LOX/COX inhibitor. Eur J Pharm Sci: Off J Eur Federation Pharm Sci. 106:231–243. 10.1016/j.ejps.2017.06.006 28599988

[CIT0013] National Research Council (NRC) 2011 Guide for the care and use of laboratory animals. 8th ed Washington, DC: The National Academies Press.

[CIT0014] Olmedo D, Marrone Paredes N, Espinosa A, Guerra Torres C, Gupta M 2016 Evaluation of the antiproliferative activity of methanolic extracts of plants from the Leguminosae family. Rev Cubana Plantas Med. 21:272–283.

[CIT0015] Organisation for Economic Cooperation (OECD). 2001. Guidelines for Testing of Chemicals 423. 2001. Acute oral toxicity-acute toxic class method. #423, Paris, France.

[CIT0016] Schetter AJ, Heegaard NH, Harris CC 2010 Inflammation and cancer: interweaving microRNA, free radical, cytokine and p53 pathways. Carcinogenesis. 31:37–49. 19955394 10.1093/carcin/bgp272 PMC2802675

[CIT0017] Shabbir A, Batool SA, Basheer MI, Shahzad M, Sultana K, Tareen RB, Iqbal J, Saeed Ul H 2018 *Ziziphora clinopodioides* ameliorated rheumatoid arthritis and inflammatory paw edema in different models of acute and chronic inflammation. Biomed Pharmacother. 97:1710–1721. 29793335 10.1016/j.biopha.2017.11.118

[CIT0018] Shi Y, Wu D, Sun Z, Yang J, Chai H, Tang L, Guo Y 2012 Analgesic and uterine relaxant effects of isoliquiritigenin, a flavone from *Glycyrrhiza glabra*. Phytotherapy Research : Ptr. 26:1410–1417. 22389128 10.1002/ptr.3715

[CIT0019] Xu N, An JF 2017 Formononetin ameliorates mast cell-mediated allergic inflammation via inhibition of histamine release and production of pro-inflammatory cytokines. Exp Ther Med. 14:6201–6206. 29250144 10.3892/etm.2017.5293 PMC5729371

